# Supplementary material for: Mediational Occupational Risk Factors Pertaining to Work Ability According to Age, Gender and Professional Job Type
Source: Int J Environ Res Public Health. 2021 Jan 20;18(3):877. doi: 10.3390/ijerph18030877 (PMC7908393; doi:10.3390/ijerph18030877)
Supplement: Supplementary file 1 [file ijerph-18-00877-s001.pdf]

Supplementary Table 1. Spearman correlation coefficients between working conditions risk variables and overall WAI scores.

Working conditions risk variables	r	p-value
Current environmental risk	-0.154	0.001
Historic environmental risk	-0.218	0.001
Current ergonomic risk	-0.292	0.001
Historic ergonomic risk	-0.340	0.001
Psychosocial risk	-0.367	0.001
Violence-discrimination	-0.286	0.001
Work-family conflict	-0.456	0.001
